# Supplementary material for: A continuum of invariant sensory and behavioral-context perceptual coding in secondary somatosensory cortex
Source: Nat Commun. 2021 Mar 31;12:2000. doi: 10.1038/s41467-021-22321-x (PMC8012659; doi:10.1038/s41467-021-22321-x)
Supplement: Supplementary file 1 — Supplementary Information [file 41467_2021_22321_MOESM1_ESM.pdf]

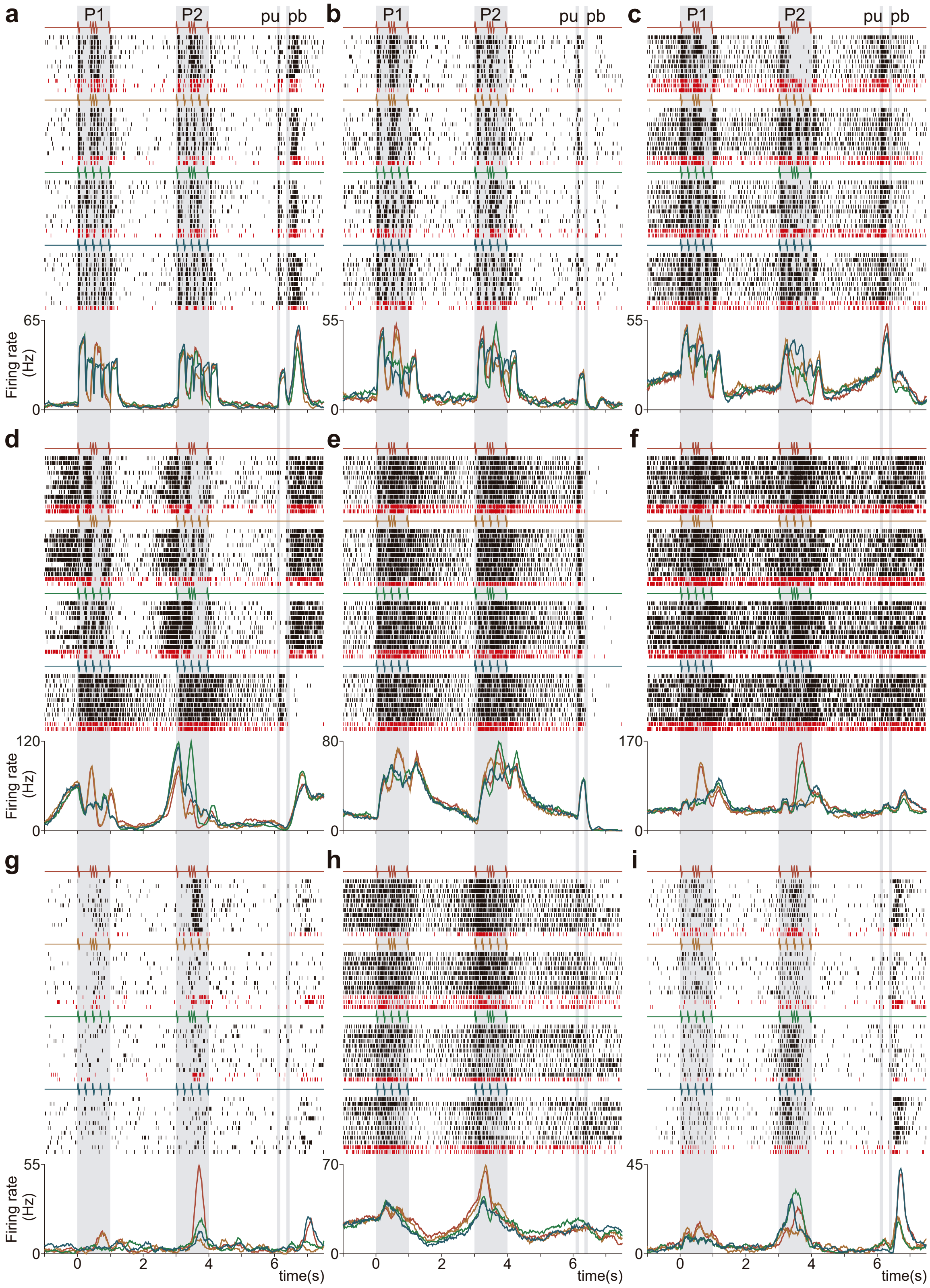

Supplementary Figure 1. Activity of single neurons in S2. S2 neurons display sensory **a-b**, intermediate **c-f** and categorical **g-i** responses during the TPDT. **a-i**) Raster plots of nine exemplary S2 neurons sorted according to the four pattern pairs of G and E stimuli, delivered during P1 and P2. Each row is one trial, and each tick is an action potential (spike). Trials were interleaved randomly, blocks are sorted by classes (only 10 out of 20 trials per class are shown). Correct and incorrect trials are indicated by black and dark red ticks, respectively. Traces below the raster plots are firing rate averages per-class [peristimulus time histograms (PSTHs)] for each neuron. Each color refers to one of the four possible pattern pairs of G and E: c1 (G-G, red), c2 (G-E, orange), c3 (E-G, green) and c4 (E-E, blue).

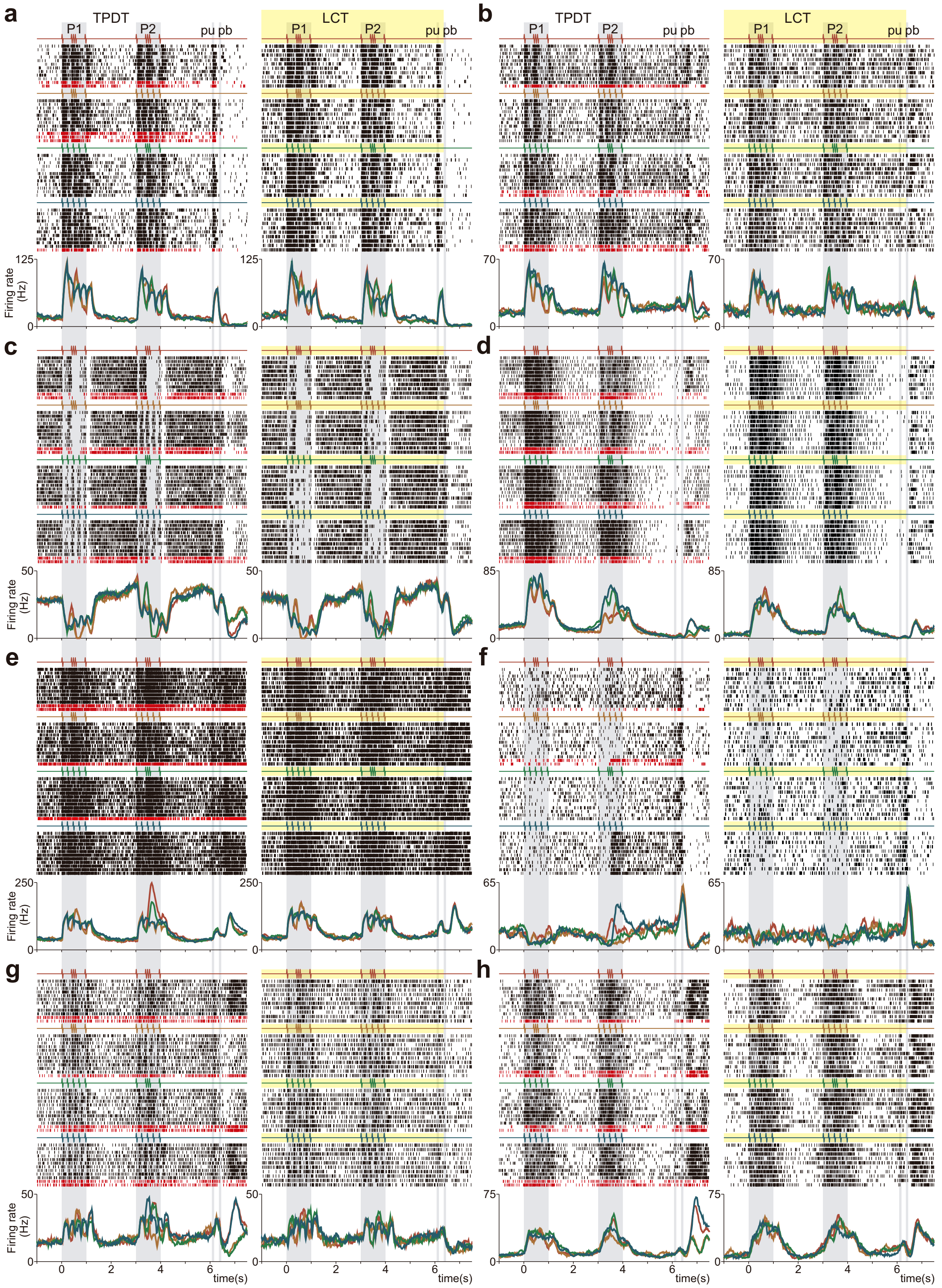

Supplementary Figure 2. Activity of single S2 neurons during the TPDT and LCT. a-f). Raster plots of eight additional exemplary S2 neurons tested in both experimental conditions: the TPDT (left) and the LCT (right). Responses are sorted according to the four possible combinations of G and E stimulus pattern delivered during P1 and P2. Correct and incorrect (only in the TPDT) trials are indicated by black and dark red ticks, respectively. Traces below the raster plots are average per-class firing rates (PSTHs) for each neuron. Each color refers to one of the four possible stimulus combinations of G and E; the resulting four classes are: c1 (G-G, red), c2 (G-E, orange), c3 (E-G, green) and c4 (E-E, blue). S2 sensory responses remained unaltered during the TPDT and the LCT (**a**, **c**). Note that the neuron in **c** depicts an invariant and inhibitory sensory response. S2 categorical responses diminished during the LCT (**d**). Decision coding after pu disappeared during the LCT (**g**, **h**). In intermediate neurons during the LCT (**b**, **d**, **e**, **g**, **h**), the sensory dynamics remain, and categorical responses vanished. Then, in intermediate neurons during the LCT, sensory responses increased and categorical coding diminished.

Class	Stimulus Pair Combinations (P1 P2)	First Stimulus (P1)	Second Stimulus (P2)	Class Selective				Decision
				Class 1	Class 2	Class 3	Class 4	
1<br>2	vs	0	1	1	1	0	0	1
1<br>3	vs	1	0	1	0	1	0	1
1<br>4	vs	1	1	1	0	0	1	0
2<br>3	vs	1	1	0	1	1	0	0
2<br>4	vs	1	0	0	1	0	1	1
3<br>4	vs	0	1	0	0	1	1	1

1 = [AUROC ≠ 0.5]   0 = [AUROC ≈ 0.5]

Supplementary Figure 3. Classification of coding responses. Coding responses were defined according to specific profiles of significant differences (1) and statistical equalities (0) between classes (ROC analysis, $p<0.05$; Methods). In this coding scheme, both 0 and 1 are important labels requiring statistical significance. Any bin that was not found to be significant received no label. Trial classes were labeled from 1 to 4 depending on the pattern combinations: c1 (red, G-G); c2 (orange, G-E); c3 (green, E-G); and c4 (blue, E-E). G denotes grouped stimulus pulses and E denotes extended stimulus pulses. Each row corresponds to the comparison of two classes, or stimulus pair combinations (1st and 2nd columns). Each binary entry indicates the result of the corresponding comparison for that row, and the binary words composed of the six digits of each column represent the possible coding profiles of the cells, based on all six possible class comparisons. The coding profiles refer to coding of the first (P1, cyan) and second (P2, green) stimulus patterns (3rd and 4th columns, respectively); coding of one specific class (5th to 8th columns, pink); and coding the decision (same [P1=P2] or different [P1≠P2]; 9th column, black). This code scheme was employed in Figs. 3a-b, 4e-h, 7e-f and Supplementary Fig. 5d-g.

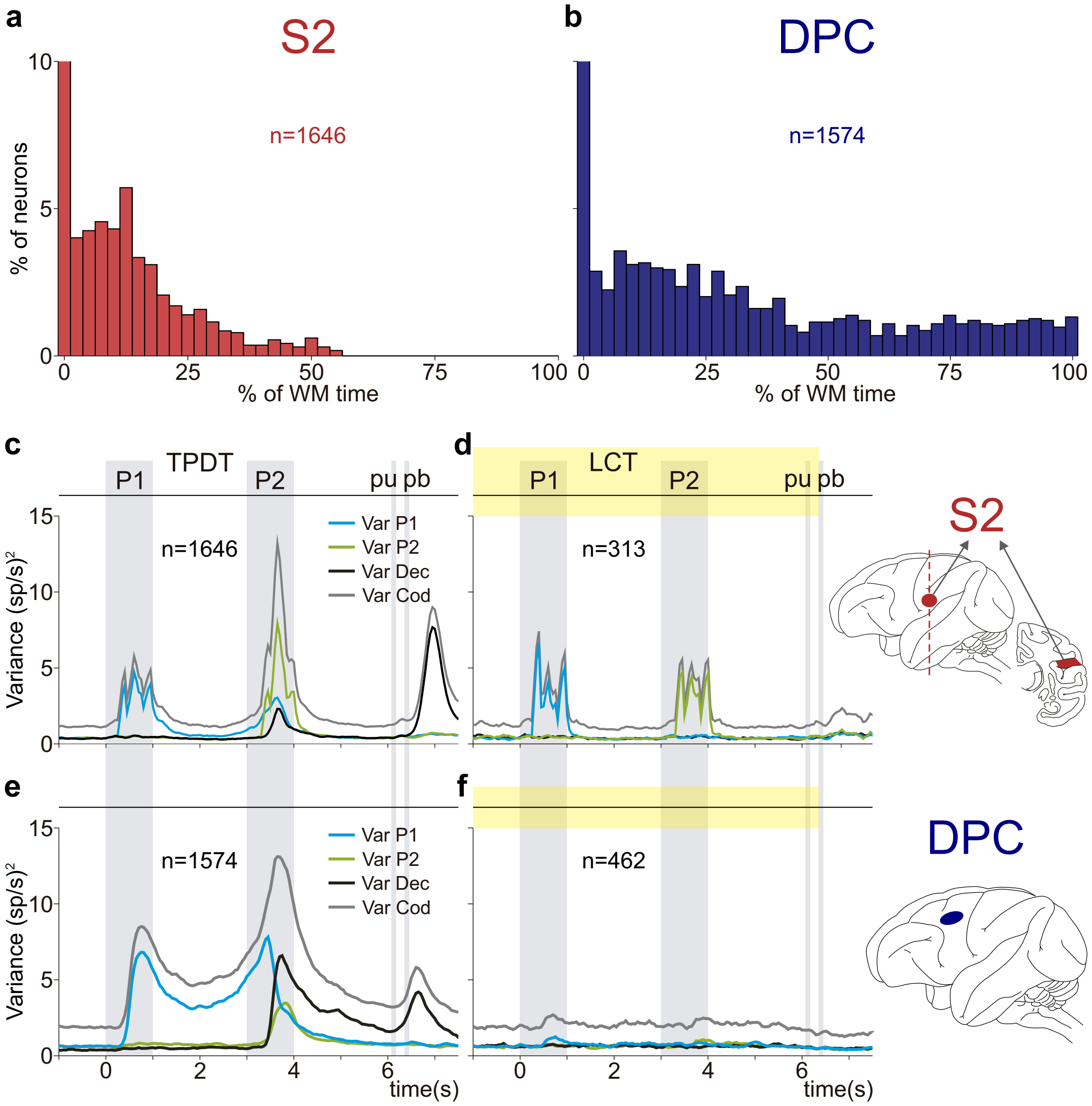

Supplementary Figure 4. Working memory coding and population variance in S2 and DPC. a-b) Histograms of the population proportions of S2 and DPC that show coding activity through the working memory delay, in percentages of time spent coding. **a**) The red histogram represents the neurons recorded for S2. Importantly, no neurons were found that code for more than 60% of the working memory delay. **b**) The blue histogram represents the neurons recorded for DPC. Around 13% of the population code for 75% of the working memory delay or more. **c-f**) One neuron could code significantly in different time bins, with disparate strength or variance. To deal with this issue, at each time bin, we calculated the population instantaneous coding variance (Var_{COD}, Eq. (1), grey) during the TPDT (S2, **c**; DPC **e**) and the LCT (S2, **d**; DPC, **f**). A greater population coding the task's parameters will yield higher values of Var_{COD}. We also calculated the population variances associated to individual task parameters in each task context: P1 (Var_{P1}, Eq. (2), cyan), P2 (Var_{P2}, Eq. (3), green) and categorical decision outcome (Var_{Dec}, Eq. (4), black). **c-d**) S2 population variances during the TPDT (n=1646) and the LCT (n=313). The population's response variability is comparable with the S2 population coding dynamics (Fig. 3a-b). **c**) The TPDT coding variance reaches its maximum value during the comparison period, where coding dynamics are most complex. Importantly, S2 Var_{COD} reveals the pure sensory responses in a clearer manner during stimulation. Furthermore, in agreement with **a**, Var_{COD} and Var_{P1} almost vanish during the middle period of the working memory. **d**) In stark contrast, Var_{COD} is only the combination of P1 and P2 stimulus identity variances (Var_{P1}, Var_{P2}) during the LCT, each restricted to its respective stimulation period. The decision outcome variance is abolished during the comparison and motor report periods. Furthermore, the maximum value of Var_{COD} reached during the LCT decreased significantly as compared to the TPDT. **e-f**) DPC variances associated to task parameters recorded during the TPDT (n=1574) and the LCT (n=462). **e**) Comparatively, DPC variance dynamics do not exhibit any abrupt peaks related to pure sensory dynamics. These results contrast markedly with those observed in S2 (**c**). **f**) DPC coding variance dynamics disappeared during the LCT, so no invariant sensory responses were found in DPC (compare with **d**). All DPC responses were subject to context-dependent modulation. We can conclude that DPC only codes fully transformed perceptual categorical signals.

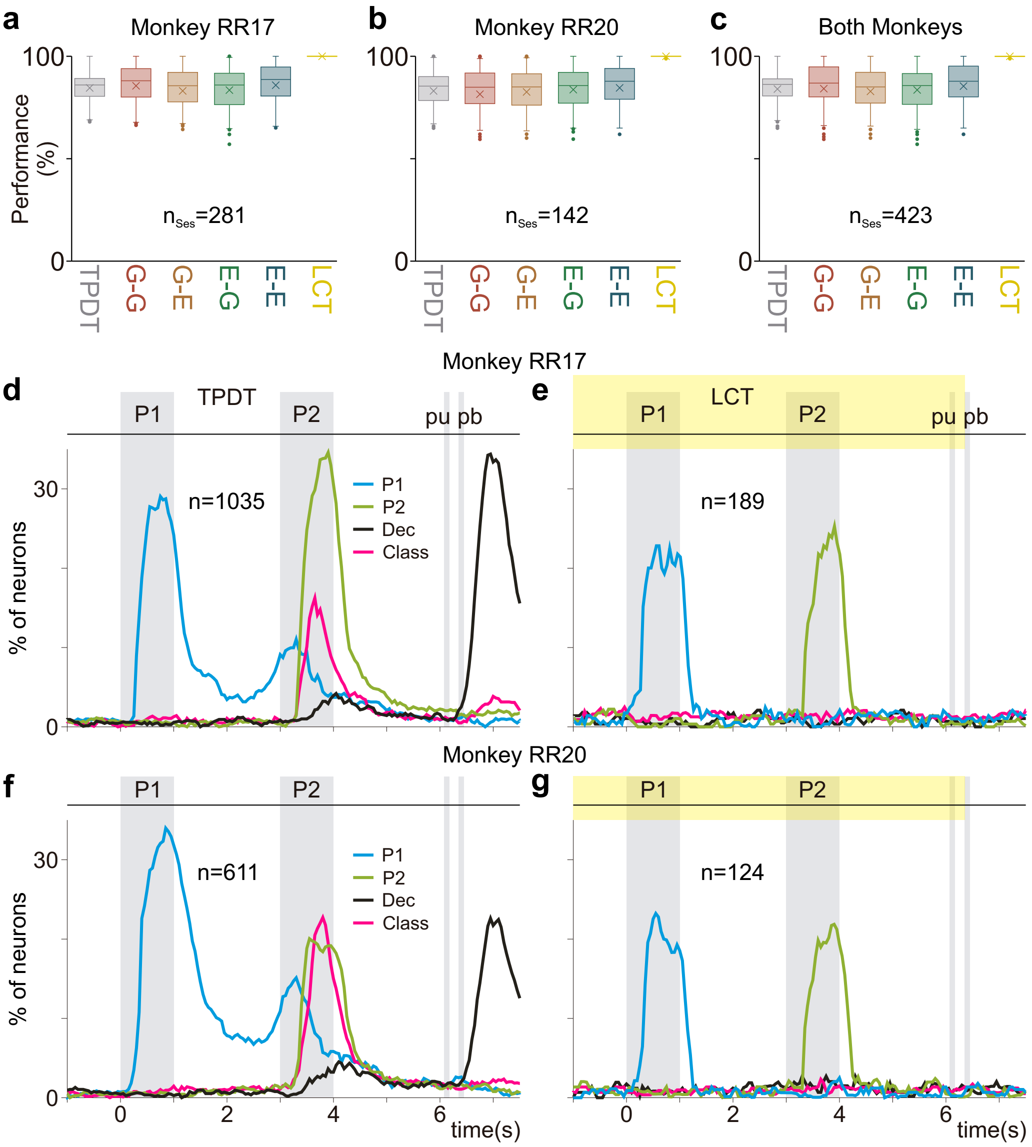

Supplementary Figure 5. Performance and population coding dynamics during the TPDT and LCT across Monkeys. a) Monkey RR17's performance for the whole TPDT (84.5%, grey, n_{SES}=281 sessions), for each class [85.6% G-G (red), 83.1% G-E (orange), 83.5% E-G (green), 85.9% E-E (blue)] and for the whole light control task (LCT) (100%, yellow, n_{SES}=49 sessions). In the LCT, the same stimuli were delivered as in the TPDT, but the rewarded push button press was visually guided. **b**) Monkey RR20's performance for the whole TPDT (83.1%, n_{SES}=142 sessions), for each class: G-G (81.5%), G-E (82.6%), E-G (83.7%). E-E (84.6%) and for the LCT (100%, n_{SES}=27 sessions). **c**) To facilitate comparison, we included performance from both monkeys' sessions (same as Fig. 1a) during the TPDT (84.0%, n_{SES}=423 sessions), G-G (84.2%), G-E (82.9%), E-G (83.6%). E-E (85.4%) and the LCT (100%, n_{SES}=76 sessions). **a-b**) Note the consistency across monkeys' performance. **d-g**) Percentage of neurons with significant coding (see Supplementary Fig. 3) as a function of time during the TPDT (Monkey RR17, n=1035; Monkey RR20, n=611) and the LCT (Monkey RR17, n=189; Monkey RR20, n=124). Traces refer to P1 (cyan), P2 (green), all class coding (pink), and decision coding (black). Despite analogous results between both monkeys during the TPDT, ranging through the comparison period, P2 coding is higher in RR17 and class coding is more prominent in RR20. In both monkeys, P1 working memory, decision, and class coding, essentially vanished during the LCT: instead, the coding was restricted to stimulus periods. Similar to DPC, all categorical and perceptual codes are abolished during the LCT, but akin to area 3b, S2 sensory responses always persist (see Fig. 7). Further, in both monkeys we identified sensory neurons (I_{Per}>0.25bits, Monkey RR17, n=71; Monkey RR20 n=34) and categorical neurons computed during the first stimulus ($I_{Is,P1}$>0.25bits, Monkey RR17, n=91; Monkey RR20, n=59) or second stimulus ($I_{Is,P2}$>0.25bits, Monkey RR17, n=116; Monkey RR20, n=68) periods. **a-c**) The box-plot statistics are all percentage values, and are listed in the following order: minimum, maximum, mean, low bound, and high bound. **a**) RR17, [TPDT: 68.9, 100, 84.5, 80.4, 89.2; G-G: 67.8, 100, 85.6, 80.1, 93.9; G-E: 67.1, 100, 83.1, 77.7, 92.1; E-G: 64.7, 99.5, 83.5, 76.4, 91.7; E-E: 65.8, 100, 85.9, 80.6, 94.8; LCT: 100, 100, 100, 100, 100]. **b**) RR20, [TPDT: 67.1, 99.4, 83.1, 78.4, 90.1; G-G: 63.9, 98.9, 81.5, 76.9, 91.8; G-E: 63.6, 99.3, 82.6, 76.1, 91.4; E-G: 64.9, 100, 83.7, 77.1, 92.1; E-E: 64.9, 100, 84.6, 79.0, 94.1; LCT: 100, 100, 100, 100, 100]. **c**) Both monkeys, [TPDT: 68.6, 100, 84.0, 80.6, 89.0; G-G: 66.2, 100, 84.2, 80.2, 94.8; G-E: 65.9, 100, 82.9, 77.1, 92.2; E-G: 64.3, 100, 83.6, 76.5, 91.6; E-E: 64.9, 100, 85.4, 80.2, 95.2; LCT: 100, 100, 100, 100, 100].

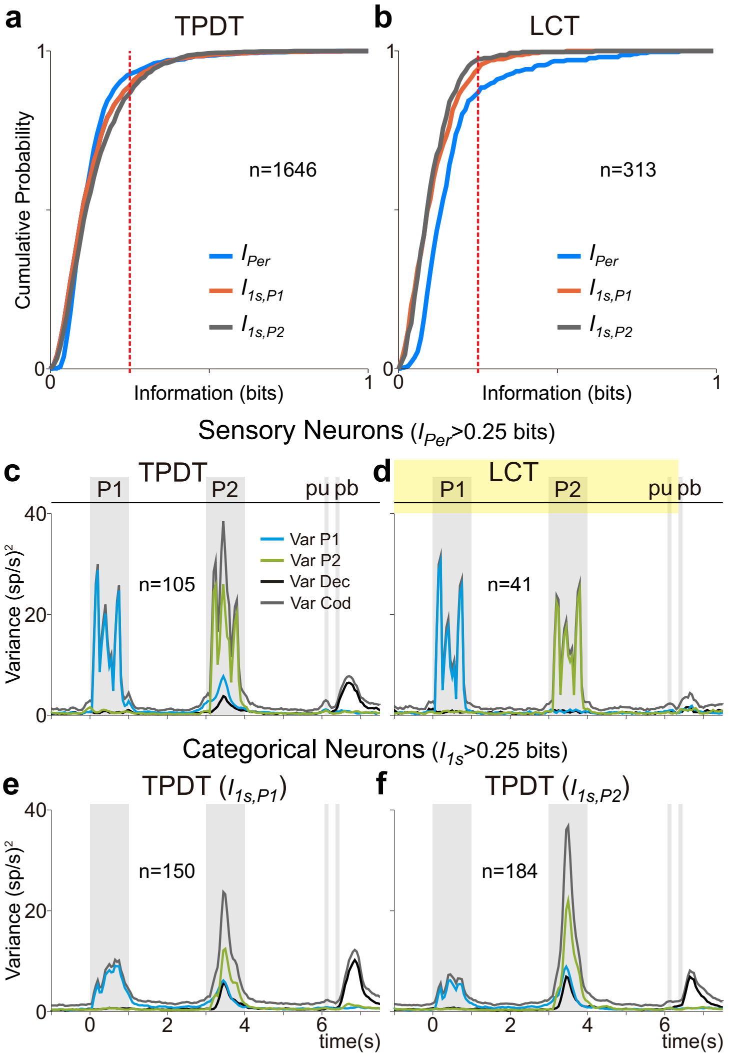

Supplementary Figure 6. Mutual information and population variance in sensory and categorical neurons. a-b) Cumulative probability distribution for periodicity (I_{Per}, blue trace, Eq. (10)) and 1s firing rate mutual information about P1 ($I_{1s,P1}$, orange, Eq. (6)) or P2 identity ($I_{1s,P2}$, grey, Eq. (6)). The value on the y axis represents the fraction of neurons with mutual information smaller or equal to the amount indicated on the x axis. **a**) Neurons recorded during the TPDT (n=1646). **b**) Neurons recorded during the LCT (n=313). A greater percentage of neurons with higher I_{Per} were found during the LCT than the TPDT. The red dashed lines indicate the mutual information criteria (I>0.25bits) used to label S2 neurons as sensory or categorical. This arbitrary boundary is useful to explore the dynamical features of the S2 neurons with higher amounts of I_{Per} or $I_{1s,P1}$. **c-f**) Population instantaneous coding variance (Var_{COD}, grey, Eq. (1)), P1 variance (Var_{P1}, cyan, Eq. (2)), P2 variance (Var_{P2}, green, Eq. (3)), and decision variance (Var_{DEC}, black, Eq. (4)) computed for S2 neuron subgroups. **c**) Sensory neurons (I_{Per}>0.25 bits, n=105) during the TPDT. Most coding variance is related to stimulus phase-locked activity. **d**) Sensory neurons (I_{Per}>0.25 bits, n=41) during the LCT. All coding variance is associated to stimulus phase-locked activity. **e-f**) Categorical neurons computed during P1 (left, $I_{1s,P1}$>0.25 bits, n=150) or P2 (right, $I_{1s,P2}$>0.25 bits, n=184). The variance associated to sensory neurons does not change based on context. On the other hand, the categorical neurons demonstrate a greater variance during the comparison than the first stimulus period.

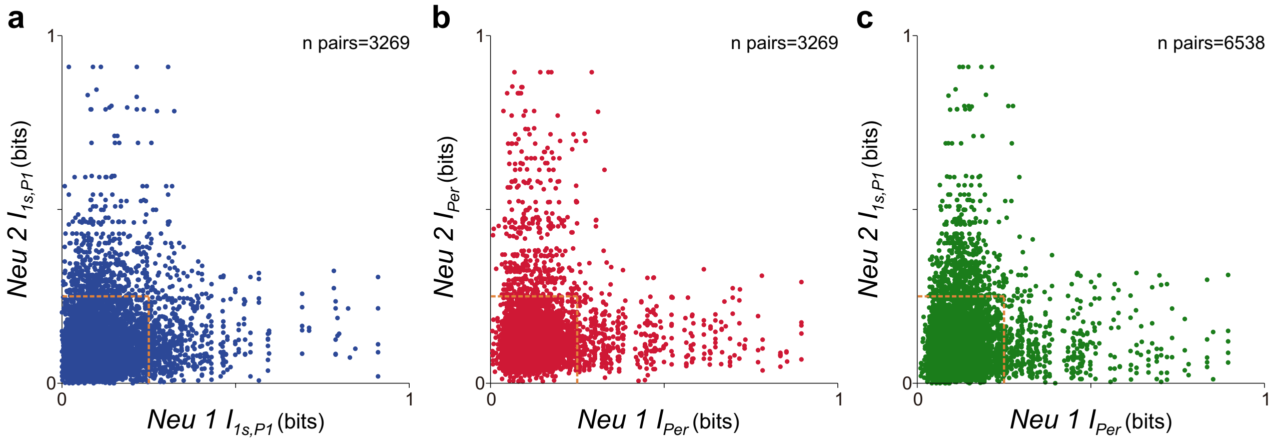

Supplementary Figure 7. Mutual information for pairs of neurons recorded together. a-c) The information values between neuron 1 and neuron 2 that were recorded simultaneously (n_{pairs}=3269 neuron pairs). **a**) The $I_{1s,P1}$ values that measure the categorical representation for neuron 1 and neuron 2. The probability of recording a pair of neurons with $I_{1s,P1}$ >0.25 bits is p=0.013 (n_{pairs}=42). The probability of recording one neuron with $I_{1s,P1}$ >0.5 bits and the other one with $I_{1s,P1}$ >0.25 bits is p=0.0024 (n_{pairs}=8). No recorded pairs had $I_{1s,P1}$ >0.5 for both neurons. **b**) The I_{Per} values used to distinguish the sensory subgroup for neuron 1 and neuron 2. The probability of recording a pair of neighboring neurons with I_{Per} >0.25 bits is p=0.0067 (n_{pairs}=22). The probability of recording one neuron with I_{Per} >0.5 bits and the other one with I_{Per} >0.25 bits is p=0.0036 (n_{pairs}=12). No recorded pairs had I_{Per} >0.5 for both neurons. **c**) The I_{Per} values of neuron 1 (x axis) plotted against the $I_{1s,P1}$ values of neuron 2 (y axis). Note that the graph is not the same if the values were to be inverted [(I^1_{Per}, $I^2_{1s,P1}$) vs. ($I^1_{1s,P1}$, I^2_{Per})], so we have twice the number of pairs (n_{pairs}=6538). The probability of recording a pair of neurons with I^1_{Per}>0.25 bits and $I^2_{1s,P1}$>0.25 bits is p=0.0078 (n_{pairs}=52). The probability to record one neuron with I^1_{Per}>0.5 bits (or $I^1_{1s,P1}$>0.5 bits) and the other one with $I^2_{1s,P1}$>0.25 bits (or I^2_{Per}>0.25 bits) is p=0.003 (n_{pairs}=19). No recorded pairs had I^1_{Per}>0.5 and $I^2_{1s,P1}$>0.5 bits. Based on these small probabilities, the different dynamics profiles cannot be spatially segregated into subnetworks. It is highly unlikely to simultaneously record neurons with similar or antagonist dynamics. All probability values were obtained from taking the number of observed occurrences and dividing by the total neuron pairs.

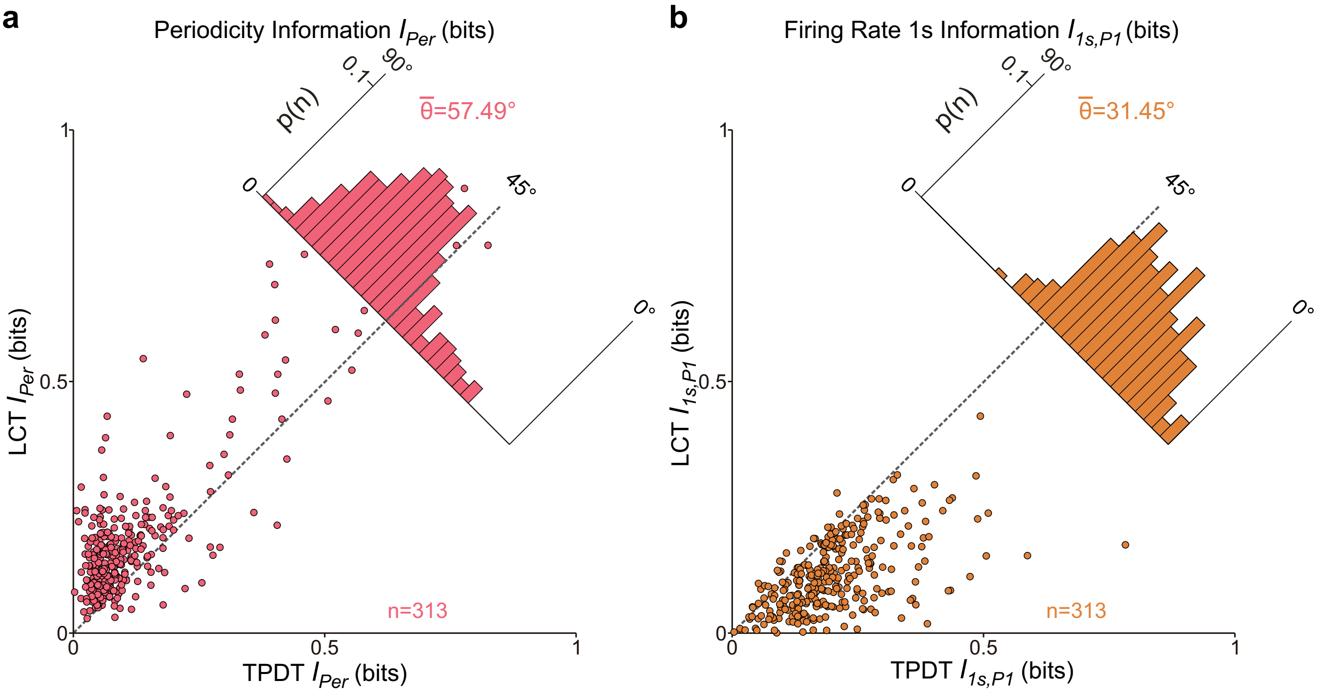

Supplementary Figure 8. Single neuron periodicity and categorical information between the TPDT and LCT. In this figure, we restricted our analysis to the neurons that were recorded during both the TPDT and LCT (n=313). Each point corresponds to one neuron tested in both tasks. Note that most of the neurons studied in this figure are intermediate, like the vast majority of the whole S2 population. Inset histograms display angular distributions of information values for each neuron. Any angle greater than 45° shows information preference for the LCT, and less than 45° shows preference for the TPDT **a**) I_{Per} in the TPDT (x axis, Eq. (10)) is compared with I_{Per} in the LCT (y axis). Angular distribution trend for these neurons (<θ>=57.49°) reflects greater I_{Per} for the LCT. **b**) $I_{1s,P1}$ in the TPDT (x axis, Eq. (6)) is compared with $I_{1s,P1}$ in the LCT (y axis). Angular distribution (<θ>=31.45°) reflects greater $I_{1s,P1}$ during the TPDT. Periodicity information grows during the non-demanding task (LCT) and categorical information increases during the cognitively demanding task (TPDT).

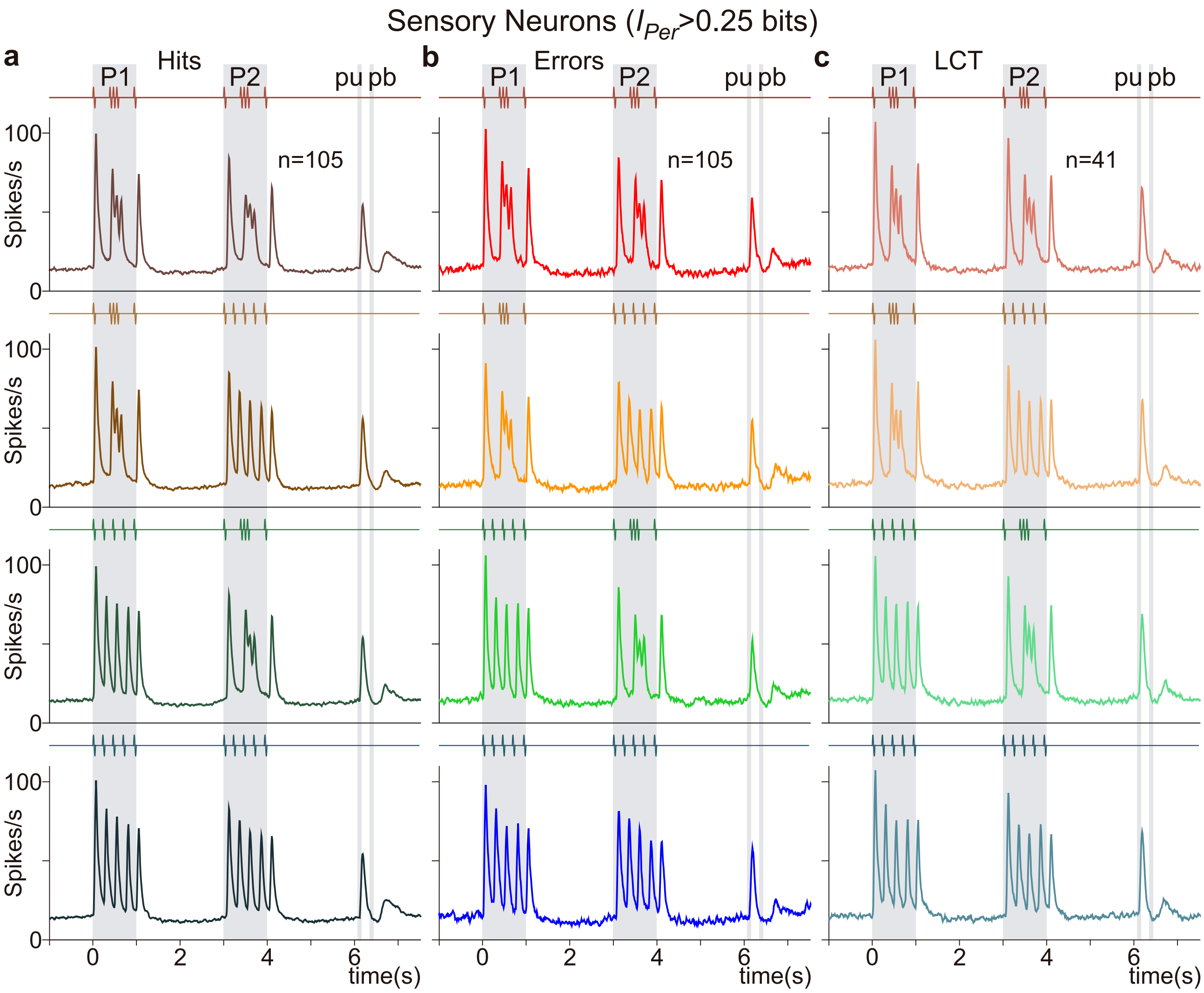

Supplementary Figure 9. Sensory responses during hit, error and LCT trials. Sensory S2 neurons selected with significant periodicity information greater than 0.25 bits (I_{Per}>0.25 bits, Eq. (10)). **a**) Normalized S2 sensory population activity for hits, **b**) errors (n=105), and **c**) LCT trials (n=41). Same color traces as in Fig. 5 for the four classes: G-G (red); G-E (orange); E-G (green); and E-E (blue). Even if the number of neurons is the same for hit and error normalized sensory population responses (n=105), the number of error trials is far fewer. For each trial class, differences between classes or conditions calculated using integral square error were found to be negligibly small (from 1.2 to 2.6%). The normalized LCT activity encompasses all trials, since no errors were found during this control.

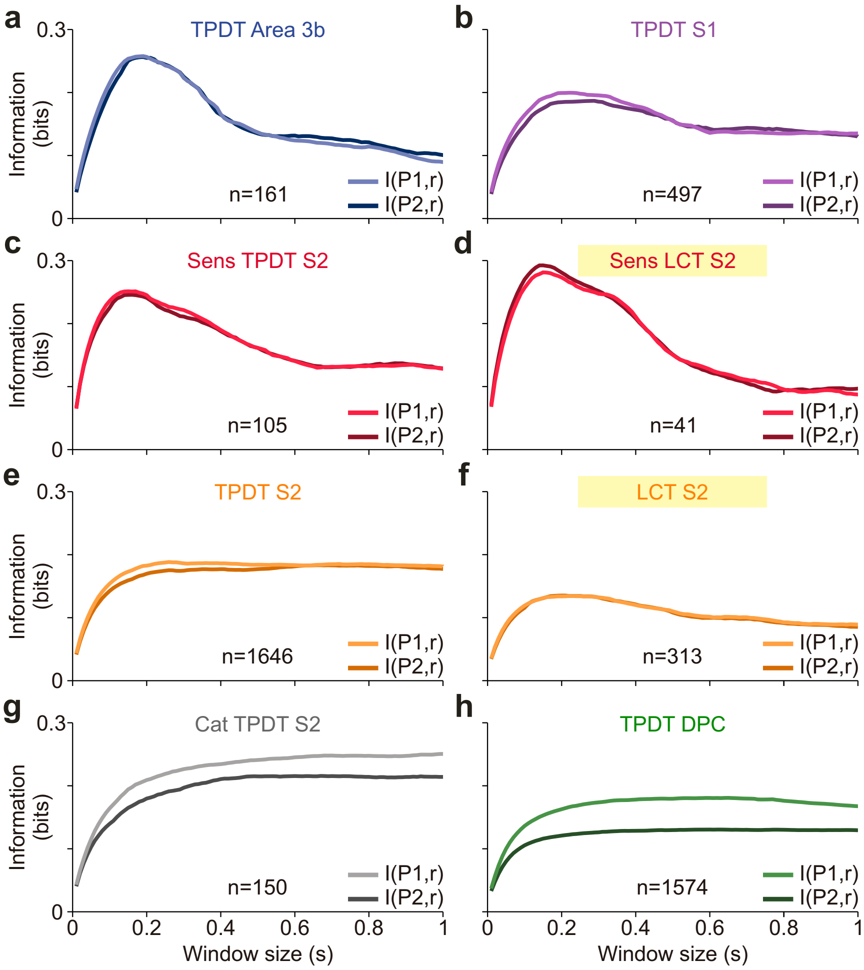

Supplementary Figure 10. Optimal integration window across cortices and subgroups of neurons. a-h) We compute the mean pattern information carried by different groups of neurons, measured in bits (Methods) as a function of window-width (10-1000ms in steps of 10ms) during P1 [light traces] or P2 [dark traces]. We calculated the mutual information (*I(P1,r)* and *I(P2,r)*) for each neuron using time windows of different lengths. Then, we averaged the information across all neurons to obtain the mean population information for each window. **a-d**) Subgroups of neurons or areas with phase-locking responses exhibit an information maximum when the window was ~200ms wide. Notably, the maximum information that can be obtained decreases significantly from area 3b (**a**) in comparison with the whole S1 population (**b**). Sensory S2 neurons (**c-d**) show comparable behavior to area 3b neurons (**a**), including a greater maximum value of mutual information as compared to S1 (**b**). Further, sensory S2 neurons exhibit higher *I(P,r)* during the LCT (**d**) than the TPDT (**c**). **e-h**) In the other populations of neurons, information reached a stationary value at around 200ms. **f**) In agreement with Fig. 3, during the LCT, information exhibited a noteworthy decrease in comparison to the TPDT (**e**). **g**) Subpopulation of categorical neurons computed during P1 (n=150). Analogous results were found with P2 categorical neurons. **h**) DPC neurons yield much less P2 mutual information. In summary, while sensory neurons exhibit a clear optimal integration window, categorical neurons reach a stationary value at around the same value, ~200ms.

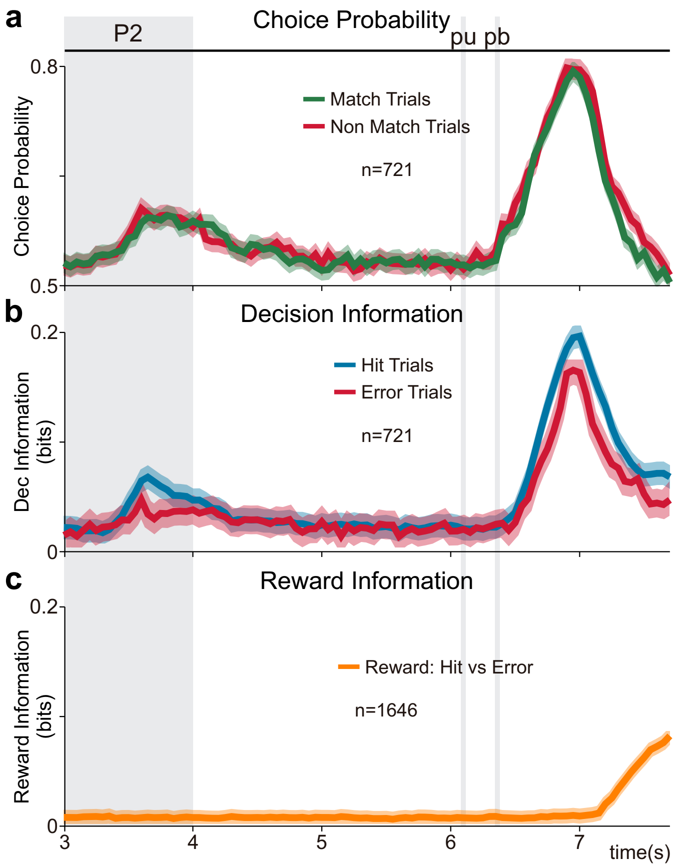

Supplementary Figure 11. Choice probability, decision and reward mutual information during hit and error trials. In this figure, we focus on the last part of the task, from 3 to 7.7s. **a-b**) To evaluate how the categorical decision signal unfolded over time, we analysed the neurons with significant decision coding (3 consecutive time bins) during hit and error trials (n=721). **a**) We computed a population choice probability index (*CP*, Methods) over time to compare the distributions for hit versus error trials in classes associated with identical decision outcomes (c1, c4 for P2=P1; c2, c3 for P2≠P1). We found similar *CP* evolution for match (P2=P1, green trace) or non-match trials (P2≠P1, red trace). Even if *CP*s reach significant values at the end of the comparison periods (*CP*~0.6), higher values were observed after the push button press (*CP*~0.8). This strong decision signal agreed with the dynamics observed in Fig. 3. **b**) We also computed the 200ms firing rate mutual information associated with the categorical decision during hit and error trials (Eq. (8), $I_{DEC}(t)$). The results were consistent with those in **a**: it is possible to decode the categorical decision identity from either hit (blue) or error trials (red). **c**) Population firing rate mutual information associated with reward computed as a function of time ($I_{REW}(t)$, Eq. (9)). We divided the z-score responses into two distributions: hit and error trials. This means that we have one distribution for the population responses during hit trials and another distribution for responses during error trials. These distributions are different from those used in **b**, where trials were first split according to the stimulus pair. S2 neurons carried significant reward information during the period after animals were rewarded for correct decision ($<t_{REW}>$=6.9s). Hence, it is possible to employ S2 activity to infer if the animal received reward or not. Note that this signal emerged subsequently to the decision signal that appeared after pb (**b**). All shadows indicate 95% confidence intervals computed through bootstrap.

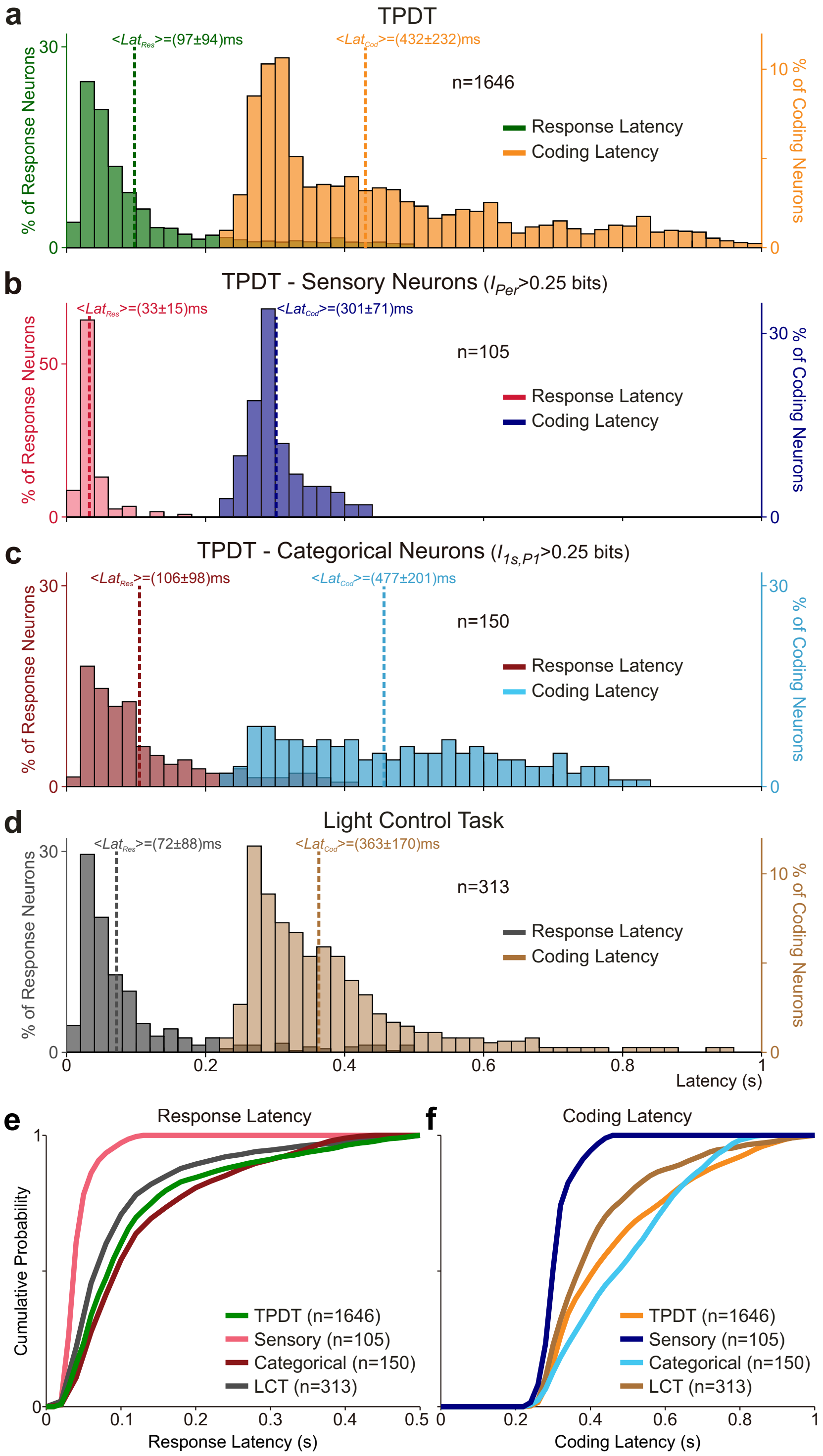

Supplementary Figure 12. Response and coding latencies in the S2 population. a-d) Response (*Lat*$_{Res}$, left axis) and coding latency (*Lat*$_{Cod}$, right axis) distributions are computed for: **a**) the whole population of TPDT neurons (n=1646); **b**) sensory neurons during the TPDT (n=105); **c**) categorical neurons during the TPDT (n=150); **d**) the whole population of LCT neurons (n=313). Mean values for each latency distribution are indicated with dashed lines (± standard deviation, S.D.). These measures allow us to estimate how long the population took to respond and code the task. Expectedly, the average coding latency across each population was longer than their corresponding response latencies. Based on experimental design, since the patterns became distinct with the arrival of the 2nd E-pattern pulse (~220ms), the coding latency must be longer than this value; the fastest coding latency across all S2 neurons was never less than 230ms. The entire S2 population responds and codes slower than the sensory subgroup (*Lat*$_{Res}$: 97ms vs. 33 ms; *Lat*$_{Cod}$: 432ms vs. 301ms, AUROC analysis, permutation test p<0.001), but faster than the categorical subgroup (*Lat*$_{Res}$: 97ms vs. 106ms; *Lat*$_{Cod}$: 432ms vs. 477ms). **e-f**) The cumulative probability distribution for *Lat*$_{Res}$ **e** and *Lat*$_{Cod}$ **f** latency for each group demonstrated in **a-d**. The value on the y axis represents the fraction of neurons with response and coding latency smaller or equal to the value indicated on the x axis. For both coding and response latencies, the sensory subgroup is the fastest and the categorical subgroup is the slowest.

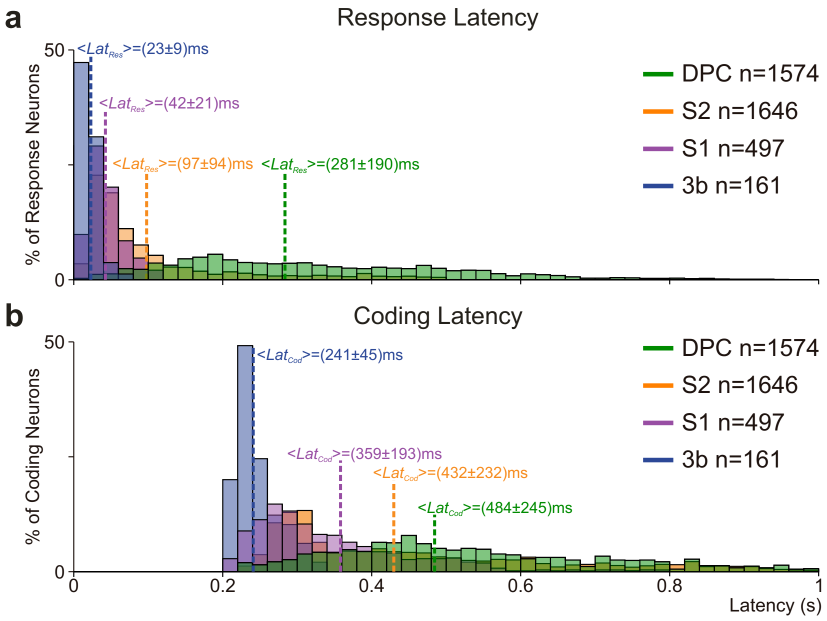

Supplementary Figure 13. Response and coding latencies across cortical areas. a-b) Response (Lat_{RES}, top) and coding (Lat_{COD}, bottom) latency distributions are computed during the TPDT for the whole DPC population (green, n=1574), the whole S2 population (orange, n=1646), the whole S1 population (purple, n=497) and the whole area 3b (S1) population (blue, n=161). Mean values for each latency distribution are indicated with dashed lines (± values indicate standard deviation, S.D.). Note that neurons from area 3b are also included in S1 population. A cortical area hierarchical order could be established in the response and coding latencies. The 3b neurons responded and coded the fastest of all (AUROC analysis, permutation test $p<0.001$), suggesting that 3b neurons constitute the earliest somatosensory input to the cortex[11,14]. However, a remarkably slight difference (AUROC $p<0.01$) distinguished the marginally higher sensory S2 Lat_{Res} and Lat_{Cod} (Supplementary Figure 12b) from the expectedly fastest 3b neuron latencies (Lat_{Res}: 33ms vs. 23ms; Lat_{Cod}: 301ms vs. 241ms). In extension, both latencies of the entire S1 population are longer than those of the S2 sensory neurons. Neurons from DPC demonstrate the longest Lat_{Res} and Lat_{Cod}, but have comparable values of coding latencies to S2 categorical neurons (Supplementary Fig. 12c, 477ms vs. 484ms, AUROC $p<0.01$); however, the S2 categorical Lat_{Res} tends to be much faster than those for DPC (106ms vs. 281ms, AUROC $p<0.001$). The implications are that since DPC coding latencies are slower, and response latencies are far slower than the slowest subset of the S2 population (categorical neurons), this categorical signal in S2 cannot originate from DPC.

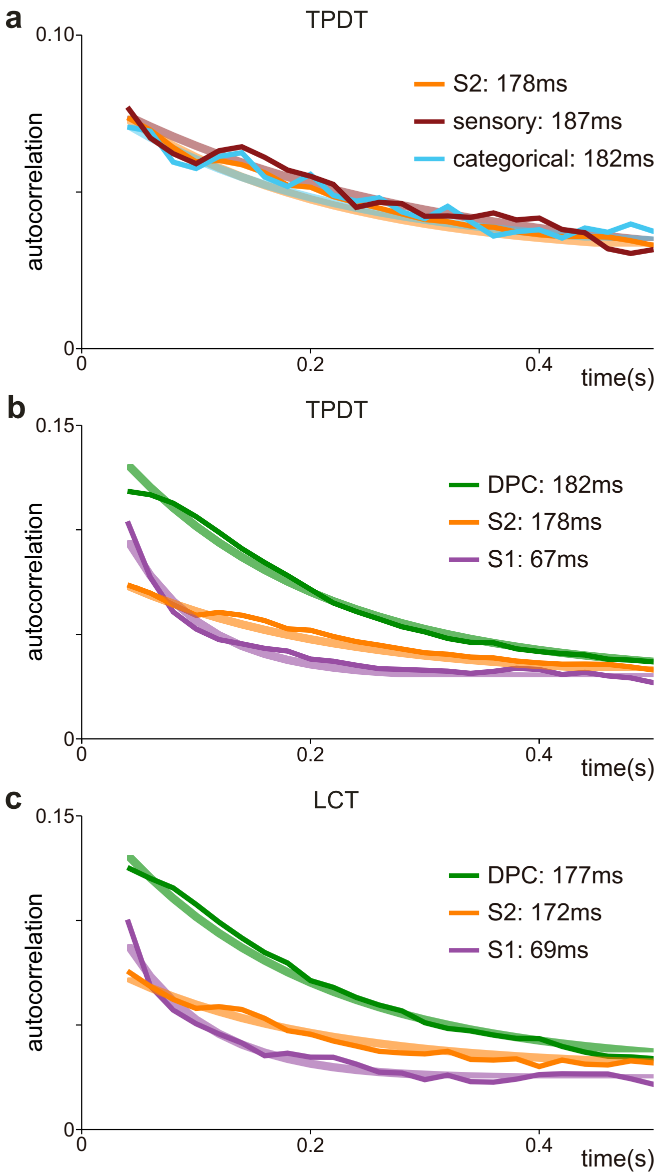

Supplementary Figure 14. Spike count autocorrelation during basal periods. The autocorrelation function was computed for neuronal activity during the basal periods for the TPDT and the LCT with 40ms time bins. An exponential decay was fit to the autocorrelation function (Eq. (11)). Confidence intervals (95%) for τ were estimated through bootstrap. Thin, darker traces show the autocorrelation values averaged across each population of neurons. Wide, lighter traces display the exponential fit for each population. **a)** Given that S2 neurons exhibit such varied dynamics, we wonder if they are part of different hierarchical processing stages. Our results of the spatial distribution of coding profiles throughout the S2 network (Supplementary Fig. 7) suggest that the continuum of sensory and categorical dynamics is part of the same network. Autocorrelation functions for the entire S2 population (orange, n=1646, τ=178 ± 10ms), S2 sensory population (brown, I_{Per}>0.25 bits, n=105, τ=187 ± 16ms) or S2 categorical population (blue, $I_{1s,P1}$>0.25 bits, n=150, τ=182 ± 14 ms). Autocorrelation was invariant for S2 and its subpopulations. Based on this and the results shown in Supplementary Fig. 7, one could intuit that even if each S2 subpopulation were engaged in antagonist roles, they are fundamentally embedded within the same cortical hierarchy. **b)** The TPDT autocorrelation function for the entire S2 population (orange, n=1646, τ=178 ± 10ms) is compared against the S1 (purple, n=497, τ=67 ± 7ms) and DPC populations (green, n=1574, τ =182 ± 5ms). Cortical areas exhibit a hierarchical order based on the autocorrelation function, demonstrating an increase in decay constant (τ) and/or autocorrelation values whilst transitioning from S1 to downstream frontal areas. Despite comparable τ values between the S2 and DPC populations, the autocorrelation results for DPC start at and maintain greater values, meaning fluctuations do not reverberate equally within the networks, despite their approximately equivalent timescales. Note that a change of ±20% in the time bin width for the firing rate calculation across areas (S1, S2, and DPC) produced no significant differences in the time constants; however, much smaller bin widths (20 ms, with steps of 10 ms) produced a rescaling of the time constants to smaller values (51, 161, and 173 ms, respectively). **c)** The LCT autocorrelation function for entire S2 population (orange, n=313, τ=172 ± 15ms) compared against the S1 (purple, n=319, τ=69 ± 10ms) and DPC populations (green, n=462, τ=177 ± 9ms). For all areas, autocorrelation functions are analogous in the TPDT and LCT. These results are further evidence that autocorrelation is a measure of an inherent feature of each cortical network that does not depend on subpopulation or context[17].
