# Supplementary material for: A continuum of invariant sensory and behavioral-context perceptual coding in secondary somatosensory cortex
Source: Nat Commun. 2021 Mar 31;12:2000. doi: 10.1038/s41467-021-22321-x (PMC8012659; doi:10.1038/s41467-021-22321-x)
Supplement: Supplementary file 3 — Reporting Summary [file 41467_2021_22321_MOESM3_ESM.pdf]

natureresearch

Corresponding author(s): Ranulfo Romo
Roman Rossi-Pool

Last updated by author(s): Feb 15, 2021

Reporting Summary

Nature Research wishes to improve the reproducibility of the work that we publish. This form provides structure for consistency and transparency in reporting. For further information on Nature Research policies, see Authors & Referees and the Editorial Policy Checklist .

Statistics

For all statistical analyses, confirm that the following items are present in the figure legend, table legend, main text, or Methods section.

n/a	Confirmed	
☐	☒	The exact sample size (*n*) for each experimental group/condition, given as a discrete number and unit of measurement
☐	☒	A statement on whether measurements were taken from distinct samples or whether the same sample was measured repeatedly
☐	☒	The statistical test(s) used AND whether they are one- or two-sided<br>*Only common tests should be described solely by name; describe more complex techniques in the Methods section.*
☐	☒	A description of all covariates tested
☐	☒	A description of any assumptions or corrections, such as tests of normality and adjustment for multiple comparisons
☐	☒	A full description of the statistical parameters including central tendency (e.g. means) or other basic estimates (e.g. regression coefficient) AND variation (e.g. standard deviation) or associated estimates of uncertainty (e.g. confidence intervals)
☐	☒	For null hypothesis testing, the test statistic (e.g. *F*, *t*, *r*) with confidence intervals, effect sizes, degrees of freedom and *P* value noted<br>*Give P values as exact values whenever suitable.*
☒	☐	For Bayesian analysis, information on the choice of priors and Markov chain Monte Carlo settings
☒	☐	For hierarchical and complex designs, identification of the appropriate level for tests and full reporting of outcomes
☒	☐	Estimates of effect sizes (e.g. Cohen's *d*, Pearson's *r*), indicating how they were calculated

Our web collection on statistics for biologists contains articles on many of the points above.

Software and code

Policy information about availability of computer code

Data collection	We employed a Cerebus (128 channels) Blackrock Microsystems PN#4176. We used a Multichannel Acquisition Processor Blackrock Microsystems. We recorded with seven array microelectrodes from Thomas Recording. We sorted the single neurons with Matlab code that we developed.
Data analysis	We developed our own codes employing C (v3.0) and MATLAB (R2020b). The code is available upon request.

For manuscripts utilizing custom algorithms or software that are central to the research but not yet described in published literature, software must be made available to editors/reviewers. We strongly encourage code deposition in a community repository (e.g. GitHub). See the Nature Research guidelines for submitting code & software for further information.

Data

Policy information about availability of data

All manuscripts must include a data availability statement. This statement should provide the following information, where applicable:

- Accession codes, unique identifiers, or web links for publicly available datasets
- A list of figures that have associated raw data
- A description of any restrictions on data availability

The data that support the findings of this study are available from the corresponding authors upon reasonable request.

Field-specific reporting

Please select the one below that is the best fit for your research. If you are not sure, read the appropriate sections before making your selection.

Life sciences study design

All studies must disclose on these points even when the disclosure is negative.

Sample size	Neuronal recordings were obtained with an array of seven independent, movable microelectrodes (2-3 MΩ 4) inserted into S2, either contralateral (left hemisphere) or ipsilateral (right hemisphere) to the stimulated hand. We collected neuronal data in blocks using different mean frequencies (5Hz, 7Hz, 15Hz). However, for the analysis described below we will focus on the neuronal responses with the stimulus set illustrated in Fig. 1A (5 Hz). In general, we recorded 20 trials per stimulus pair (c1; c2; c3; c4). Recording sites changed from session to session; the locations of the penetrations were used to construct surface maps in S1, S2 and DPC by marking the edges of the small chamber (7 mm in diameter) placed above each area. Sample sizes per mean frequency were the total number of recorded sessions for each. Sample sizes used in all analyses were determined as the number of single neurons identified by sorting, across all recording sessions for stimuli with a mean frequency of 5Hz.
Data exclusions	All single neurons identified during the sorting process were included in all the analysis. We did not exclude any recorded single neuron. All experiments performed have been noted in this summary. The stimulus blocks with varying mean frequencies that were not considered had 33 sessions with a mean of 7Hz and 32 sessions with a mean of 15Hz, recorded across both monkeys. Since we recorded 432 sessions for the block of 5Hz mean frequency, we chose to focus on the largest data set possible.
Replication	Our neural data was recorded from two different monkeys. Similar results were found in both animals in the same sample cortical areas: secondary somatosensory cortex.
Randomization	Monkeys were trained to perform the task, randomizing the stimulus pairs. Then, at the beginning of each trial the animals did not know which stimulus pair will be delivered.
Blinding	Recording sites changed from session to session and no bias was introduced by the experimental recording and data analysis.

Reporting for specific materials, systems and methods

We require information from authors about some types of materials, experimental systems and methods used in many studies. Here, indicate whether each material, system or method listed is relevant to your study. If you are not sure if a list item applies to your research, read the appropriate section before selecting a response.

Materials & experimental systems

n/a	Involved in the study
☒	☐ Antibodies
☒	☐ Eukaryotic cell lines
☒	☐ Palaeontology
☐	☒ Animals and other organisms
☒	☐ Human research participants
☒	☐ Clinical data

Methods

n/a	Involved in the study
☒	☐ ChIP-seq
☒	☐ Flow cytometry
☒	☐ MRI-based neuroimaging

Animals and other organisms

Policy information about studies involving animals; ARRIVE guidelines recommended for reporting animal research

Laboratory animals	We employed to trained Rhesus Macaque (Macaca Mulatta) from the colony of the Institute of Cellular Physiology. Experiments were performed in two adult male (6–11 years old) rhesus macaques, ranging from 6 to 10 kg.
Wild animals	Our study did not involve wild animals.
Field-collected samples	Our research did not involve samples collected from the field.
Ethics oversight	Monkeys were handled according to the institutional standards of the National Institutes of Health and Society for Neuroscience. All protocols were approved by the Institutional Animal Care and Use Committee of the Instituto de Fisiología Celular of the National Autonomous University of Mexico (UNAM).

Note that full information on the approval of the study protocol must also be provided in the manuscript.
